# Fibroblast Growth Factor 21: A Fascinating Perspective on the Regulation of Muscle Metabolism

**DOI:** 10.3390/ijms242316951

**Published:** 2023-11-29

**Authors:** Shuo Li, Jun Chen, Panting Wei, Tiande Zou, Jinming You

**Affiliations:** Jiangxi Province Key Laboratory of Animal Nutrition, Jiangxi Agricultural University, Nanchang 330045, China; lishuo199x@163.com (S.L.); junchen@jxau.edu.cn (J.C.); 18679954991@163.com (P.W.)

**Keywords:** FGF21, muscle metabolism, stress response, insulin signaling, myogenic development

## Abstract

Fibroblast growth factor 21 (FGF21) plays a vital role in normal eukaryotic organism development and homeostatic metabolism under the influence of internal and external factors such as endogenous hormone changes and exogenous stimuli. Over the last few decades, comprehensive studies have revealed the key role of FGF21 in regulating many fundamental metabolic pathways, including the muscle stress response, insulin signaling transmission, and muscle development. By coordinating these metabolic pathways, FGF21 is thought to contribute to acclimating to a stressful environment and the subsequent recovery of cell and tissue homeostasis. With the emphasis on FGF21, we extensively reviewed the research findings on the production and regulation of FGF21 and its role in muscle metabolism. We also emphasize how the FGF21 metabolic networks mediate mitochondrial dysfunction, glycogen consumption, and myogenic development and investigate prospective directions for the functional exploitation of FGF21 and its downstream effectors, such as the mammalian target of rapamycin (mTOR).

## 1. Introduction

Muscle system homeostasis is maintained through a complex regulatory network, which is a crucial basis for sustainable growth and development and imperative to life activities and physiological signals [1]. The highly plastic properties of a muscle result from its quality and strength and are susceptible to gene mutations, disease states, and hereditary or acquired factors. The pathological symptoms of muscle disorders include muscle atrophy, muscle weakness, and muscle mitochondrial dysfunction, which further lead to muscle fiber damage, muscle inflammation, and other muscle myopathies [1,2,3,4]. The increasing incidence of muscle-associated metabolic disorders has become an urgent medical problem for maintaining a wide range of muscle health [4,5]. Thus, elucidating the intrinsic regulatory mechanisms of muscle metabolism may establish a characteristic therapeutic approach for alleviating muscle homeostasis imbalance.

FGF21 is a secreted protein discovered by Japanese scientists in the liver and thymus of mice at the beginning of this century [6]. Consistently, it was also detected in the pancreas, adipose tissue, skeletal muscle, and other organs, which are important for metabolic homeostasis [7]. The function of FGF21 was initially found to be focused on the regulation of glucose uptake by adipocytes through increasing cellular metabolism to alter the presence of blood glucose and improve insulin sensitivity [7,8]. In addition, FGF21 can also reduce lipid accumulation, promote lipid oxidative metabolism, and diminish the occurrence of lipid metabolic syndrome [9,10]. With the development of modern studies, it has been found that skeletal muscle is also one of the secretory tissues and effector organs of FGF21, and the performance of FGF21 in various aspects of skeletal muscle metabolic homeostasis has been emphasized [11,12]. The abnormal expression and secretion of muscle-derived FGF21 usually reflect physiological changes in muscle that are speculated to be the cause of metabolic disorders, such as changes in the muscular membrane potential status, mitochondrial dysfunction, and muscular dystrophy [13,14,15].

As a myogenic regulator, FGF21 is closely related to muscle metabolism [16,17]. FGF21 is induced as an endocrine factor and promotes the formation of oxidized types of muscle fibers during myogenic differentiation in C2C12 myoblasts [18]. Moreover, the induction of muscle-derived FGF21 as an adaptive adjustment measure distally regulates insulin signaling, muscle fiber development, energy metabolism homeostasis, obesity, and other systemic problems to improve metabolic characterization [19,20,21,22]. However, the effect of FGF21 depends on the dose response, and moderate induction and acute induction will lead to different consequences; thus, FGF21 can be used as a therapeutic agent or a marker of a certain disease [23,24]. Compared to wild-type mice, chronic alcohol-reared FGF21-knockout mice exhibit metabolic abnormalities such as cardiac damage and cardiac dysfunction, indicating that FGF21 is a protective agent against alcohol-induced cardiomyopathy [25].

To elucidate the effect of FGF21 on the regulation of muscle metabolism homeostasis and the development of myopathy, we provide a brief explanation of the biological structure and physiological and pharmacological effects of FGF21, focusing on the metabolism-related effector genes and proteins of FGF21 in the muscle stress response, insulin signaling, and muscle development. Finally, the role of FGF21 in muscle metabolism is summarized. This review focuses on assessing the significance of FGF21 in regulating muscle metabolic homeostasis and explores the advantages of FGF21 in the prevention and treatment of muscle disorders.

## 2. FGF21-Mediated Regulation of Metabolism Networks

**Figure 1:**
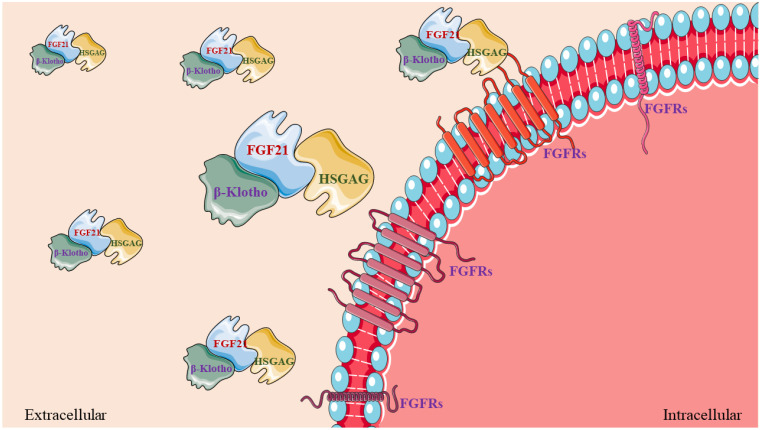
FGF21, β-Klotho, and HSGAG form a trimer complex and bind to the FGFR (FGFR1 or FGFR4) on the cell membrane.

The fibroblast growth factor (FGF) family consists of six subfamilies, among which the capability of the FGF19 subfamily to act in an endocrine manner distinguishes it from five other subfamilies with paracrine signaling [26]. The FGF19 subfamily members are also hormone factors secreted by skeletal muscle, and these family members have similar physiological functions, such as modifying skeletal muscle metabolism [27,28]. The three members of the FGF19 subfamily, FGF19, FGF21, and FGF23, exhibit decisive effects in the bile acid metabolism, glycolipid metabolism, and phosphate homeostasis systems in specific approaches, respectively [26,29]. The main reason for this particular channel is that FGF19 subfamily members have a low affinity for the FGF receptor (FGFR), and FGF19 subfamily members alone cannot produce a stabilizing effect by binding to FGFR, which usually requires the assistance of heparan sulfate glycosaminoglycan (HSGAG) and Klotho (e.g., α-Klotho or β-Klotho) [26,28]. The premise condition for binding to FGFR1 or FGFR4 is that FGF21 connects to HSGAG at the amino terminus and β-Klotho at the carboxyl terminus to form a trimeric complex [26,28,30] (Figure 1).

Research evidence suggests that the area of action of FGF21 is mainly concentrated in fat, liver, and muscle and is accompanied by the source-pleiotropy and action-pleiotropy of this bioactive factor [31,32]. Multiple studies have shown that FGF21 can alleviate insulin resistance, but interestingly, the synergistic effect of FGF21 and insulin is not negligible [33]. In addition, the role of FGF21 in fatty acid metabolism, liver ketogenic metabolic balance, and skeletal muscle homeostasis also should not be ignored [34,35]. Extensive biological research has highlighted that FGF21 can alleviate nonalcoholic fatty liver disease formation and promote the browning of white adipose tissue [36,37]. Similar studies have also demonstrated that muscle-derived FGF21 induced by muscle mitochondrial stress mediates the energy-dissipating response of brown adipose tissue through the phosphorylation of eukaryotic initiation factor-2a (elF2a) and prevents lipid accumulation in mice [38]. In addition, FGF21 is likely a regulator that reverses the decrease in muscle mass caused by obesity or inflammation and improves muscle mass under some special physiological conditions [39]. FGF21 has shown a remarkable ability to regulate intramuscular fat development and seems to be a prospective target for meat quality control, such as inhibiting the upregulation of genes related to lipid synthesis (e.g., peroxisome proliferator-activated receptor-γ, sterol regulatory element binding proteins 1) [40,41].

**Figure 2:**
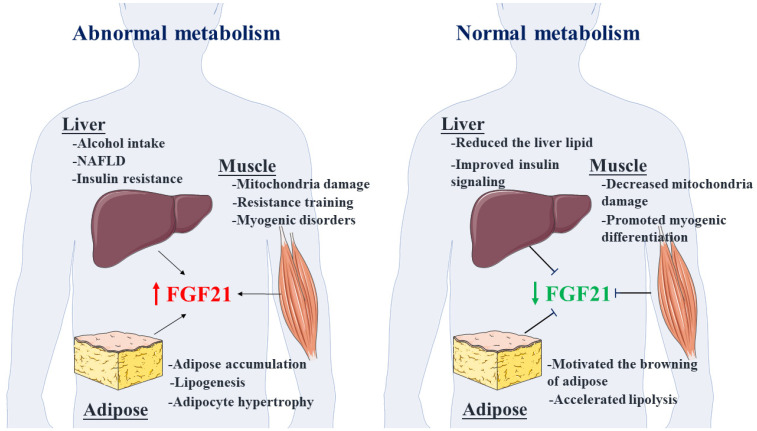
FGF21 effectively regulates the systemic abnormal metabolism induced by exogenous and endogenous stimulation. When the metabolism of the liver, muscle, and adipose tissue of the body is abnormal, the secretion of FGF21 is increased, and the elevated FGF21 can alleviate the abnormal metabolism of the liver, muscle, and adipose tissue and maintain metabolic homeostasis.

Previous research results have shown that part of the circulating FGF21 is also secreted by skeletal muscle [27]. FGF21 is induced upon muscle membrane depolarization through activation of extracellular ATP signaling, specifically the P2YR/PI3K/Akt/mTOR signaling pathway [14]. FGF21 can also be detected in myopathy caused by various mitochondrial abnormalities, including mitochondrial translation failure, mitochondrial DNA deletion, and impaired respiratory chain conduction [24,42]. Consistently, clinical experiments have also demonstrated that FGF21 and growth differentiation factor 15 (GDF15) are potential markers of mitochondrial dysfunction, muscle dysplasia, and genetic disorders deficient in oxidative phosphorylation [43,44]. In addition, exercise, as a potent inducer of FGF21, can regulate energy status and myosin synthesis in skeletal muscle through mTOR and AMP-activated protein kinase (AMPK) to ameliorate the occurrence of obesity and muscle development [21,45]. According to the molecular structure, action target, and functional pleiotropy of mTOR, there are two compound forms, namely, the mammalian target of rapamycin complex 1 (mTORC1) and the mammalian target of rapamycin complex 2 (mTORC2) [46,47,48]. Among them, mTORC1 has abundant biochemical functions, including regulating protein synthesis, lipid metabolism, muscle development, insulin signaling, mitochondrial damage, energy metabolism, and sensing changes in nutrients [49,50,51]. These results also demonstrate that the regulation of mTORC1 signaling in skeletal muscle is a potential action target through FGF21 and its downstream factors. Most currently available data revealed that an exercise-induced increase in FGF21 also reduces myocardial fibrosis and alleviates myocardial function by inhibiting the expression of fibrosis factors. In addition, FGF21 promotes a positive trend in myocardial energy metabolism via the transduction of Sirtuin 1 (Sirt1) signaling and preserves the normal biological function of the heart. Additionally, FGF21 can alleviate autophagy and apoptosis induced by osteoarthritis by mediating Sirt1-mTOR signaling [52,53,54]. More importantly, FGF21 improves the proportion of oxidized muscle fibers in skeletal muscle, alleviates oxidative stress damage to skeletal muscle mitochondria, reduces the generation of skeletal muscle myopathy, and better maintains muscle metabolic homeostasis [18,55,56]. However, it should be noted that many studies established the sensitivity characteristic of FGF21 for protein content, and the production of FGF21 is induced when protein intake is reduced or the amount of synthesis is insufficient, likely resulting in decreases in weight, muscle mass, and vitality [47,57,58]. For example, higher circulating FGF21 may be associated with sarcopenia and poor muscle mass in elderly individuals [59]. Moreover, in an amyotrophic lateral sclerosis (ALS) model, the activation of the FGF21 signaling pathway slowed the onset of ALS by regulating the exercise capacity, metabolic status, and inflammation levels of mice [60]. The above briefly summarizes the physiological roles of FGF21 in overall metabolism, including regulating the metabolic capacity of muscle and adipose tissue (Figure 2). In the following sections, we will elaborate on the effects of FGF21 in alleviating muscle stress, improving muscle glucose metabolism, and regulating muscle development.

## 3. Muscle Stress Response

Several observations have confirmed that FGF21 is a protein factor related to the stress response that may act on mTORC1, activating transcription factor 2 (ATF2), activating transcription factor 3 (ATF3), activating transcription factor 4 (ATF4), ERK1/2, and p38 to compose a stress-response network to protect the body from stress injury [61,62,63]. As recently summarized, the myogenic endocrine factor FGF21 is always produced in large quantities in response to muscular stress, such as ER stress, mitochondrial dynamic anomalies, and obstruction of the respiratory chain [62,64,65]. A considerable amount of myogenic circulating FGF21 not only acts on skeletal muscle but also acts as a director to regulate specific physiological metabolism. For example, myogenic FGF21 improves mitochondrial damage and the integrated stress response [38,66]. A classic research experiment also demonstrated that the aberrant expression of mitochondrial uncoupling protein 1 in skeletal muscle caused a comprehensive stress response, accompanied by a significant increase in muscle FGF21 and β-Klotho, enhanced fatty acid oxidative metabolism, and decreased muscle mass [67]. However, it was surprising that there was no functional loss or autophagy in skeletal muscle, and this result was also validated by the interference of uncoupling agents with C2C12 myoblasts [67]. These findings led to investigations on how mammalian FGF21 is involved in regulating muscle metabolism. Subsequent studies have revealed that mammalian FGF21 indeed critically regulates metabolic homeostasis in muscle, especially in the context of stresses, such as muscle mitochondrial dysfunction and endoplasmic reticulum stress [68,69].

### 3.1. FGF21 Alleviates Muscle Mitochondrial Dysfunction

The continuous fusion and division of mitochondria meets the energy requirements of muscle, maintains the normal distribution and stability of mitochondria, and optimizes the mitochondrial dynamic control system [65,68,70]. Mitochondrial stress or mitochondrial hyper-mitosis and the resulting induction of apoptosis activate the mitochondrial unfolded protein response and comprehensive stress response [68], leading to muscle injury [71]. FGF21 protects mitochondria from abnormal division by inhibiting protein molecules that mediate mitochondrial division, thereby preventing cell apoptosis and maintaining the normal physiological function of muscle [72]. Impaired mitochondrial function results in the release of reactive oxygen species (ROS), and with the assistance of ATF4, myotube FGF21 is massively induced as an endocrine regulator [73,74]. An experiment in skeletal muscle cells also demonstrated that mitochondrial dysfunction is a prominent factor in the induction of FGF21 and described FGF21 transcription and expression [13]. ATF2 specifically binds to the proximal FGF21 promoter to activate FGF21 transcription. Likewise, the myogenic active transcription factor myogenic differentiation antigen (MyoD) also participates in the pre-transcriptional modification of FGF21 with the assistance of p38MAPK during this process. More importantly, the expression of ROS is significantly increased during mitochondrial stress, which may change the energy state of cells, and then muscle cells release FGF21 through the perception of internal cellular energy state [13,74]. Moreover, the ATF4-mediated amino acid response element (AARE) responds to improve the energy state by driving the mitochondrial folate cycle, inducing purine metabolism, establishing the dNTP pool, and reshaping the one-carbon metabolic pathway [61,75]. Subsequently, further experiments also revealed a specific activation channel between comprehensive mitochondrial stress and FGF21, and the core reaction is mediated by the phosphorylation of elF2a. The complete stress response in the muscle endoplasmic reticulum activates the production of protein kinase R-like ER kinase (PERK) and the phosphorylation of elF2a and ultimately induces FGF21 through ATF4 transcriptional transduction [38]. Consistently, this result was demonstrated using a small molecule activator of elF2a phosphorylation in the transcription and translation of FGF21 [38].

The depletion of FGF21 generates significant cardiomyopathy in mice, which provokes widespread cardiac dysfunction and subsequent induction of a decline in the global longitudinal strain and ejection fraction [69]. Severe mitochondrial dysfunction and decreased optic atrophy-1 (OPA-1) levels are induced in FGF21-knockout mice [69]. OPA-1 plays an important role in maintaining the stability of mitochondrial DNA, the mitochondrial respiratory chain complex, and the mitochondrial membrane potential [23]. FGF21 overexpression provides negative feedback on mitochondrial damage by inducing recovery of the mitochondrial dynamics and thereby ameliorating myocardial mitochondrial damage, and these effects may be related to the regulation of OPA-1 by FGF21-FGFR1 signaling [69]. A further convincing experiment revealed that FGF21 plays a considerable role in myocyte respiratory chain dysfunction [13]. The abnormal respiratory chain function of myocytes results in mitochondrial diseases and aberrant energy indexes. The mechanism by which FGF21 improves mitochondrial metabolism is related to the activation of mTOR, YY1, and PGC-1α under the mediation of PI3K/Akt. Akt and p70S6K are the upstream and downstream effector points of mTORC1, respectively, with phosphorylation activity increasing significantly [55]. Although the numerous research findings described above have shown that PERK, ATF2, ATF4, AARE, GDF15, ROS, and elF2a exert a protective effect in biochemical reactions as opposed to mitochondrial stress in muscle, the prominent and authoritative finding is that FGF21 can be considered a biomarker of mitochondrial stress and the comprehensive muscle stress response [76].

mTORC1 is a prospective comprehensive regulator of muscle mitochondrial stress syndromes [77]. The abnormal metabolism of mitochondrial DNA or the mitochondrial respiratory chain will markedly activate the mTORC1 signal and generate a series of mitochondrial stress syndromes through ATF4, such as mitochondrial myopathy, imbalance of muscle mitochondrial folate metabolism, and impaired one-carbon metabolism [61,78]. These abnormalities are due to an excessive FGF21 content in plasma, and rapamycin acts as a repressor to improve the mitochondrial stress response, protecting skeletal muscle from mitochondrial damage, promoting the generation of red muscle fibers in skeletal muscle with mitochondrial damage, and thus improving skeletal muscle metabolism [61]. The related mechanism may involve a reduction in the FGF21 content by inhibiting mTORC1 signaling and downregulating the phosphorylation of S6 and S6K [61]. Therefore, FGF21 may act as a messenger and a marker in the regulation of mTORC1.

**Figure 3:**
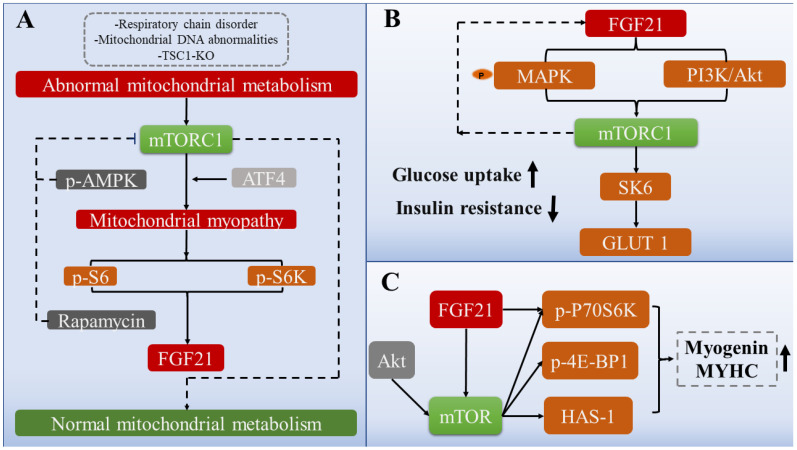
FGF21-mTOR signaling network and physiological roles. (**A**) The major signaling pathways of mTORC1 improve abnormal mitochondrial metabolism. (**B**) The major signaling pathways of mTORC1 improve insulin resistance. (**C**) The major signaling pathways of mTOR promote myogenic development.

Interestingly, AMPK likely mediates energy metabolism and is antagonistic to mTORC1 in regulating muscle mass and protein production [79,80]. Consistent with this occurrence, it was confirmed that the induction of AMPK phosphorylation in chicken skeletal muscle leads to the impaired transduction of mTORC1-related signaling pathways, including blocked protein synthesis and restricted phosphorylation of S6K1, 4E (eIF4E)-binding protein 1 (4E-BP1), and S6 ribosomes [81]. Based on the current research results, FGF21 induction may rely upon AMPK expression, and FGF21 is also critical to the physiological functions of mTORC1 [82]. However, AMPK and mTORC1 have opposite effects in some aspects. The presence of AMPK is likely a prerequisite for the induction of FGF21 in impaired muscle mitochondrial fatty acid oxidation, and the induced FGF21 reduces the phosphorylation of mTOR (Ser2448), a critical regulatory point of mTORC1 activity, and the phosphorylation of P70S6K [82]. Therefore, whether FGF21 acts as an intermediate bridge regulating the relationship between mTORC1 and AMPK and maintaining muscle homeostasis remains controversial. A myriad of studies have demonstrated that the activity of mTORC1 has a certain rhythm, and the high tide and low ebb phases alternate [83,84]. Only by correctly inducing the activation of mTORC1 can the potential effect be fully realized. The fact that acute induced mTORC1 activation promotes protein synthesis and contributes to muscle hypertrophy, whereas chronic mTORC1 activation caused by TSC1 depletion has the opposite effect would support the above assertion [49] (Figure 3A).

### 3.2. The Role of FGF21 in Endoplasmic Reticulum Stress in Muscle

The endoplasmic reticulum acts as the primary organelle for muscle protein synthesis and secretion and also clears misfolded proteins, of which SEL1L is the complex that regulates the control of misfolded proteins [85]. The depletion of SEL1L in the skeletal muscle of wild-type mice generates a significant endoplasmic reticulum homeostasis imbalance, which provokes widespread compensatory increases in protein synthesis and enhancements in protein degradation and FGF21 secretion [85]. Subsequently, FGF21 partially regulates systemic metabolism, and the underlying mechanism may be that SEL1L loss activates the integrated stress response in skeletal muscle and acts in the eIF2⍺–ATF4 axis [85]. ATF4 is always accompanied by the emergence of a comprehensive stress response and seems to be a biomarker of endoplasmic reticulum stress [86]. This effect, in turn, promotes the secretion of a large amount of FGF21, suggesting a crucial mediating role in ameliorating skeletal muscle oxidation and relieving endoplasmic reticulum stress [75,87]. Exogenous inducers stimulate rat cardiomyocytes to activate different degrees of endoplasmic reticulum stress, resulting in the increased expression of proteins related to cardiomyocyte injury [88]. The overexpression of FGF21 alleviates cardiomyocyte stress by increasing the phosphorylation of FGFR1 and ERK1/2, but the effect of FGF21 is abolished when the phosphorylation of FGFR1 and ERK1/2 is inhibited in a moderately stressed rat cardiomyocyte model. Although such observations demonstrate that FGF21 may be the fundamental regulator of the damage response, the effect of FGF21 will disappear when cardiomyocytes are subjected to high-intensity stress [88]. These findings imply that FGF21 may be secreted only by self-regulation, and its impact has certain limitations. In a critical illness mouse model, the muscular endoplasmic reticulum is severely stressed; however, compared with FGF21-knockout mice, non-FGF21-knockout mice show more moderate ER stress [17]. Moreover, exogenous FGF21 supplementation does not improve muscle strength or cellular stress in mice with critical illness [17].

**Figure 4:**
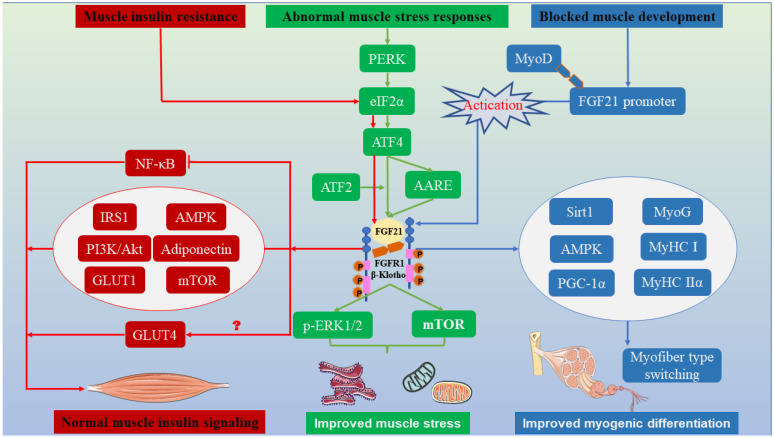
Summary of the effects of FGF21 on the regulation of muscle metabolism and development. Metabolic disorders result in the increased secretion of endogenous FGF21 in the muscle. Part of the circulating FGF21 will act on mitochondria and the endoplasmic reticulum to alleviate mitochondrial damage and endoplasmic reticulum stress or promote the transformation of muscle fiber type and further accelerate glucose uptake in the muscle.

Under specific physiological conditions, mTORC1 signaling is activated and induces ER stress in skeletal muscle, such as in a tuberous sclerosis complex 1 (TSC1) gene-knockout model. FGF21, as a critical stress molecule, is secreted in large quantities, and this secretion is accompanied by modifications of overall metabolism, including changes in skeletal muscle integrity and muscle development and differentiation [89,90]. Moreover, a subsequent study demonstrated that mTORC1 is activated and leads to increases in abnormal mitochondrial numbers and proapoptotic factors in the skeletal muscle of TSC1-deficient mice [89]. This effect results in mitochondrial dysfunction, skeletal muscle oxidative stress, and muscle fiber contraction by stimulating signal transducer and activator of transcription 3 (STAT3)-GDF15 signaling, but the oxidative metabolism and muscle fiber development of skeletal muscle are modified by constraining of mTORC1 activity [89]. In addition, FGF21 is involved in the endoplasmic reticulum stress response mediated by S-adenosine methionine and homocysteine and promotes lipid metabolism [91,92]. The above-described research results fully demonstrate the functional pleiotropy and pathway diversity of myogenic FGF21 for muscle mitochondrial performance. These findings specifically elaborate on how FGF21 ameliorates mitochondrial stress and muscle endoplasmic reticulum stress through multiple signaling pathways and specifically introduce the role of mTOR in FGF21-mediated response systems (Figure 4).

## 4. Muscle Glucose Metabolism

Higher levels of circulating FGF21 concentrations related to a high incidence of type 2 diabetes have been observed in mammals [93], which is analogous to how insulin infusion contributes to the upregulation of FGF21 expression in human skeletal muscle, indicating that FGF21 may be a myocyte factor involved in the regulation of glucose metabolism [34]. These findings led to investigations on how mammalian FG21 is involved in regulating muscle insulin signaling. Subsequent studies have revealed that mammalian FGF21 significantly regulates glucose metabolism in the muscle [66,73,94], especially in insulin-dependent and insulin-independent regulation.

### 4.1. FGF21 Preserves Muscle Glucose Metabolism in an Insulin-Dependent Manner

Skeletal muscle is the primary organ harboring pathways through which the endocrine factor is involved in energy regulation, glycogen conversion, and glucose uptake. Muscle-derived FGF21 can positively regulate skeletal muscle insulin signals to maintain whole-body blood glucose stability and energy balance [66,73]. Subsequent studies have also demonstrated that circulating FGF21 is closely related to muscle insulin resistance, and FGF21 levels are increased under conditions of hyperinsulinemia or decreased insulin sensitivity in muscle [34,95]. Moreover, as one of the most effective insulin sensitizers to date, the injection of FGF21 significantly reduces the blood glucose levels in *ob*/*ob* and diet-induced obese mice [96,97]. However, the function of FGF21 is affected to varying degrees when insulin resistance or insulin signal transduction disorder occurs [98]. For instance, the concentration of FGF21 in plasma significantly increases under conditions of type Ⅰ myotonic dystrophy and insulin resistance [99]. Under normal physiological conditions, FGF21 effectively promotes p-β-Klotho, p-ERK, p-FGFR, and p-FGS2α expression, activating downstream targets and correcting abnormal metabolism in the body [99]. However, when secondary or primary dysfunction occurs in skeletal muscle mitochondria with severe external stimuli (e.g., palmitate or excessive obesity), the signal transduction of FGF21 is severely damaged and even inhibits the expression of p-β-klotho, p-ERK, p-FGFR, and p-FGS2α, ultimately reducing glycogen uptake, and even worse, causing myotube loss [100,101]. The main reason for this is that palmitic acid is thought to induce insulin resistance by inhibiting insulin signaling and activating protein kinases. Meanwhile, palmitic acid inhibits FGF21 signaling by down-regulating β-Klotho and reducing p-FGFR [101]. However, in another palmitic-acid-induced insulin-resistance model, FGF21 still plays an important role in improving insulin signaling, but with the help of other molecular regulation. Dimethyl itaconate (DITA) alleviates palmitic-acid-induced insulin resistance in C2C12 myocytes by stimulating AMPK/FGF21/PPARδ signaling [102]. These studies indicate that in some special cases, FGF21 alone cannot achieve the expected effect, and other factors are needed to assist.

The findings described above suggest that FGF21 may be a compensatory secretion factor that counteracts the metabolic challenges caused by insulin resistance. A controlled trial found that FGF21 promotes glucose transport in the extensor digitorum longus muscle and soleus muscle of patients with type 2 diabetes to compensate for decreased insulin sensitivity [103]. This hypothesis may be valid because exogenous FGF21 activates a series of practical measures that maintain energy homeostasis, such as the expression enhancement of β-Klotho, FGFR3, and glucose transporter type 4 (GLUT4) and the increased efficiency of ketone metabolism and myogenic activity (e.g., elevated myogenin) [104]. Further investigation revealed the intrinsic physiological mechanism of FGF21 in improving glucose metabolism. This may be because FGF21 increased the phosphorylation of insulin signaling-related proteins (e.g., insulin receptor substrate 1 (IRS1) and Akt), inhibited the activation of NF-κB inflammatory factors, promoted glycogen conversion (e.g., glucose transporter type 1 (GLUT1) and GLUT4), and elevated the expression of β-Klotho in the skeletal muscle myotube insulin-resistance model constructed by exogenous drugs [105]. A similar phenomenon of increased β-Klotho has also been observed in adiponectin-mediated FGF21 improvement of blood glucose [106]. Another study revealed that adiponectin, as a downstream effector of FGF21, enhances Akt activation and promotes glucose clearance in the soleus and gastrocnemius muscles of mice because adiponectin is one of the sensitizing regulators of insulin [106,107]. The enhanced insulin sensitivity observed with FGF21 treatment has been attributed to its biological property of acting as a signal transduction terminal for exogenous substances to improve insulin signaling. Subsequent studies further confirmed that this substance promotes the secretion of FGF21 from muscle cells by activating the general control nonderepressible 2 (GCN2)-eIF2α-ATF4 pathway in vitro. Concomitantly, the significance of FGF21 has also been demonstrated by the mouse growth phenotype and expression of related proteins (e.g., FGF21 and Akt) [108]. Although the results describe how FGF21 promotes glucose metabolism by regulating insulin sensitivity, another interesting study found that in the presence of insulin, low concentrations of FGF21 enhance the effect of insulin on glucose uptake, whereas high concentrations of FGF21 completely inhibit glucose uptake; thus, the underlying mechanism needs to be further investigated [109].

### 4.2. FGF21 Preserves Muscle Glucose Metabolism in an Insulin-Independent Manner

Although the insulin-dependent mechanism has been widely observed, the insulin-independent strategy has been indicated to improve glucose homeostasis in muscle. Studies on mouse 3T3-L1 and primary human adipocytes have shown that FGF21 regulates blood glucose metabolism by increasing glucose uptake independently of insulin [94]. Xiang et al. demonstrated that manipulating glycolysis and promoting glycogen transformation by regulating gene expression and glucose receptor protein in skeletal muscle, which contributes to glucose disposal, activates the glucose metabolite sensor carbohydrate-response element-binding protein and acts on FGF21 [110]. In addition, energy homeostasis is also closely related to insulin signal transduction genes [111]. Subsequent research confirmed these results and further suggested that the system can perceive an internal energy deficiency through AMPK and induce the production of FGF21 with the assistance of Akt1 when mitochondrial fatty acid oxidation is impaired [82]. FGF21 can inhibit the serine phosphorylation of IRS1 and promote the tyrosine phosphorylation of IRS1 in insulin signaling [82]. The latter contributes to insulin signaling and increases glucose uptake by skeletal muscle through GLUT1 signaling, thereby improving the energy pool [82]. In addition, FGF21 mediates energy homeostasis regulation through PI3K/Akt signaling in the case of mitochondrial metabolic damage, which may contribute to the compensatory metabolism for the loss of mitochondrial energy regulation by FGF21 [55]. Of note, GLUT4 translocation and PI3K/PKCξ signaling are considered the determining effects of FGF21-mediated glucose homeostasis [109].

mTOR and mTORC1, as metabolic regulatory proteins, have a wide range of effects. One of the most credible studies systematically illustrates the role of FGF21-mTORC1 signaling in glucose metabolism. The phosphorylation of mitogen-activated protein kinase by FGF21 mediates activation of mTORC1/SK6 signaling and promotes the expression of the downstream effector gene GLUT1, which promotes glucose uptake and alleviates insulin resistance [112]. Interestingly, this signal forms a negative feedback loop to reverse and stimulate FGF21 expression [112]. A similar observation has shown that an mTORC1 signaling-specific knockout in skeletal muscle results in a higher plasma glucose content and systemic insulin resistance compared with wild-type mice. These results indicate that mTORC1 signaling in skeletal muscle is quite critical for glucose clearance [90]. Because FGF21 can reduce insulin resistance, there is also an interesting study that does not focus on how FGF21 alleviates insulin signaling pathways but instead focuses upstream to modify FGF21 and construct FGF21 analogs through protein engineering to enhance its functionality and perdurability [113]. The application of this effort has achieved initial results in mice, and it significantly improved the role of FGF21 in glucose uptake and insulin sensitization [113]. The first indication that plant chemicals can also be efficient FGF21 mimics was also demonstrated by Rebollo-Hernanz et al. [114]. These mimics can act as effective agonists of the FGF21 receptor to improve ERK1/2 phosphorylation and promote mTOR signaling [114] (Figure 3B). Interestingly, insulin signaling in muscle is also linked to changes in liver and fat. The depletion of PTEN in hepatocytes of mice significantly induces FGF21, improves muscle insulin sensitivity, and leads to decreased obesity, indicating a certain synergistic effect among the liver, muscle, and fat [115]. Another study found that myogenic FGF21 improves glucose tolerance and insulin sensitivity through the browning of white fat [19]. In summary, this chapter elaborates on the role of FGF21 in the glycogen signaling pathway and glucose metabolism in skeletal muscle, focuses on the proteins and genes involved in FGF21-mediated glucose metabolism, and finally demonstrates the criticality of FGF21 in skeletal muscle glucose metabolism (Figure 4).

## 5. Muscle Development

Muscle plays a fundamental role in metabolism, promoting heat production, maintaining normal metabolic activities, and increasing the efficiency of energy conversion to glycogen [116]. Moreover, it is also an important endocrine tissue and the target of certain hormones and factors, which are irreplaceable for the regulation of systematic metabolism [117,118]. Experimental investigation shows that myogenic differentiation is a strictly programmed process of self-growth that may be affected by many factors, including internal physiological effects and external environmental effects [119]. Ribas et al. found that FGF21 is a crucial driving element in the myoblast differentiation of mouse C2C12 and rat L6 cells [13]. According to the results, a large amount of FGF21 was expressed when myocytes were differentiated into myotubes, and this result was also verified in human LHCN-M2 cells [13]. This research suggests that FGF21 can be produced by skeletal muscle and can promote skeletal muscle development and myogenic differentiation. Similar to myoblast differentiation, FGF21 signaling is critical for protecting against muscle atrophy via the autophagy–lysosomal pathway [20]. These results indicate that the function of mammalian FGF21 in muscle physiology has been investigated in various physiological contexts involving muscle development.

### FGF21 Regulates Multiple Pathways to Mediate Myogenic Differentiation

FGF21 affects many cellular processes and signaling pathways. Recent observations have demonstrated that the expression of FGF21 is positively correlated with the degree of myogenic differentiation in C2C12 myocytes [120]. With the continuous increase in FGF21 expression, the myogenic factors MyoD and myogenin (MyoG) showed the same trend. Detection of the mRNA expression of myosin heavy chain Ⅰ (MyHC Ⅰ) and myosin heavy chain Ⅱα (MyHC Ⅱα) revealed that FGF21 drives the transformation of muscle fibers from the glycolysis type to the oxidation type through the TGF-β1-Smad2/3-MMP9 and p38 MAPK signaling pathways and continuously promotes the formation and extension of myotubes [121]. These results have also been replicated in mice [18]. Liu et al. observed that the primary mechanism involved is the binding of MyoD to the FGF21 promoter to activate transcription and translation, and Sirt1/AMPK/PGC-1α signal transduction assists mice in completing myogenic differentiation and muscle fiber type transformation [18].

With the weakening of muscle strength, the plasma FGF21 content continues to rise, and excessive FGF21 will stimulate oxidation in the body, which helps to increase muscle metabolism [122]. Under the conditions of poor nutrition (e.g., fasting), the mitochondrial energy supply is limited to nutrient deprivation, the FGF family is stimulated and exhibits differential expression, and these expression patterns will change based on differences in sex and skeletal muscle type [11]. Research has shown that in the fasting state, FGF21 levels in the plasma of male mice were increased 11.9-fold compared with the normal physiological condition, while in female mice, FGF21 levels were increased 23.2-fold. A similar tendency also occurred in different skeletal muscles. Fasting increased FGF21 expression in the soleus muscle of female mice and decreased the expression of the FGF activation marker gene fibronectin leucine-rich transmembrane protein 2 (FLRT2) in the tibialis anterior muscle; however, no such trend was observed in the soleus muscle [123,124]. Apart from this, the expression levels of other FGF family factors and lipid metabolism-related genes (e.g., FGF2, FGF15, FGF23, peroxisome proliferator-activated receptor-α, and carnitine palmitoyl transferase 1 α) were also different, and this test also fully confirmed the space specificity of gene expression [123,124]. In addition, fasting also inhibits respiratory chain conduction and the tricarboxylic acid cycle, including curbing succinate dehydrogenase and cis-aconitase, and intensively stimulates the expression of FGF21 [11]. Studies have shown that the conduction process of the mitochondrial respiratory chain depends on various coenzymes (e.g., Fe-S complexes); when Fe-S coenzyme deletion forms Fe-S deficiency syndrome, which is marked by a deficiency of succinate dehydrogenase and cis-aconitase, the expression of ketogenic enzymes (HMGCS2 and BDH1), PGC-1α and FGF21 is promoted [11]. Additionally, the proportion of type Ⅰ muscle fibers, which are mainly oxidized, is significantly increased in skeletal muscle and facilitates the oxidative function of skeletal muscle [11]. This secretion pattern of FGF21 can be reproduced when the respiratory chain of skeletal muscle cells is inhibited in vitro [11,123]. However, a small problem likely needs to be considered through the above series of experimental studies. Whether there is a correlation between the increase in oxidized muscle fibers and the increase in FGF21 induced by Fe-S coenzyme deletion needs to be further verified. In addition, the relationship between the number of cytochrome C oxidase-negative muscle fibers and plasma FGF21 content can also be used to infer whether the mitochondrial respiratory chain is defective [125]. Studies have shown a positive correlation between them, and the former is associated with mitochondrial respiratory chain defects [125]. In addition to skeletal muscle, FGF21 can inhibit smooth muscle cell calcification by inhibiting the activity of alkaline phosphatase or alleviating vascular oxidative stress, which are conducive to improved muscle development [126,127].

Although FGF21 can promote the myogenic expression of C2C12 cells, the overexpression of FGF21 likely has the exact opposite effect on myogenic metabolism [128]. Another study showed that the overexpression of FGF21 decreases the cross-sectional area of muscle fibers and the total muscle mass [129]. This finding is mainly observed because the total number of muscle fibers does not show an apparent change, and the main factor determining the muscle fiber area is the protein synthesis rate of muscle fibers [129]. In FGF21-knockout mice, the rate of protein synthesis is higher than that of oxidation, and the expression of BCL2-interacting protein 3 (Bnip3) is inhibited [130]. Bnip3 is a pro-apoptotic protein, and the inhibition of Bnip3 expression downregulates autophagy, thereby protecting against muscle atrophy and total muscle loss [130]. However, under the condition of FGF21 overexpression, the protein synthesis rate is less than the protein oxidation rate, which stimulates the expression of Bnip3 protein and acts on FGF21, resulting in a reduction in the muscle fiber area and muscle atrophy [129]. In this study, Bnip3 was determined to be the critical element in the regulation of whether muscle loss occurs in the presence of FGF21 [129,131]. In addition, an exciting point worth further exploration is that FGF21-knockout mice have a “fasting advantage”. This is mainly reflected by the fact that the knockout of FGF21 in mice increases the storage of muscle energy, protects muscle from autophagy injury, and promotes the generation of type IIb muscle fibers [129]. Therefore, regarding muscle fiber and muscle development, FGF21 in skeletal muscle may also be used as a stress-response factor to judge the total muscle volume.

As reviewed above, we outlined the role of mTOR in mitochondrial oxidative stress and insulin signaling in skeletal muscle. The following sections will focus on FGF21-mTOR-mediated muscle development. Studies have shown that mTOR has a prominent capacity to assist skeletal muscle in the fight against muscle atrophy and increase muscle mass in adaptation to load-bearing or leisure [132]. The underlying mechanism may be the activation of the Akt/mTOR signaling pathway and its downstream target genes and proteins, including p70S6K and HAS-1/4E-BP1, effectively promoting muscle enlargement and inhibiting degradation [83]. Consistent with the above results of mTOR mediation, administration of an mTOR inhibitor reduced myotube development and muscle hypertrophy trends and reduced the synthesis of downstream target genes and proteins, including MYHC and MyoG [133]. In addition, FGF19 can stimulate the phosphorylation of S6K1 and ERK1/2 in skeletal muscle, and the former is the main target of mTORC1 to govern myogenic cell development [134,135]. A similar observation has been made between FGF21 and FGF19, since FGF21 and FGF19 are from the same subfamily and have similar biological effects [31]. The expression of FGF21 and β-Klotho in the muscle of piglets with intrauterine growth retardation was increased [132]. Nevertheless, muscle quality and myogenic ability were significantly decreased, as determined by the expression of related myogenic proteins and genes [132]. Furthermore, the phosphorylation of mTORC1 and S6K1 was downregulated [132]. However, β-Klotho knockdown enhanced myogenesis and upregulated mTORC1 and S6K1 phosphorylation. This mechanism may indicate that FGF21 activates β-Klotho and affects muscle development [132] (Figure 4).

## 6. Conclusions and Perspectives

Based on a myriad of studies, the biological effects of FGF21 in organismal conditions and the regulation of metabolism at all levels to maintain homeostasis are now distinct [136]. Over the last few decades, this field has gained momentum due to a deep understanding of both FGF21 biochemical function and metabolic pathways to constantly explore the underlying mechanisms of how FGF21 regulates metabolism, such as stress responses, insulin resistance, and myogenic development in muscle. More importantly, FGF21 may improve muscle mitochondrial dysfunction and aberrant insulin signaling through a mechanism linked to complex regulatory networks, which include mTOR, ATF2, ATF4, GLUT1, and GLUT4. While the roles of FGF21 in muscle metabolism could provide clues to the mechanism of action in regulating muscle development and its downstream pathways, this should only be considered a superficial phenomenon to reference FGF21 effects. Therefore, to a large extent, an in-depth exploration of unknown factors, such as mTOR and its derived regulatory units, would further our understanding [46].

The main focus of FGF21 research will be to determine whether the cellular mechanism can explain the surface phenomenon of FGF21 in muscle metabolic disorders. Although some mechanisms have been elucidated through mTORC1 and its downstream targets S6K1 and 4E-BP1, the scope of action is limited. For example, the focus of the interaction between FGF21 and mTORC1 has chiefly been on alleviating oxidative stress in skeletal muscle, but little research has been conducted on its relation to the regulation of insulin signaling and promotion of myogenic development [137]. Specifically, the crosstalk between muscle-derived FGF21 induction and the regulation of mitochondrial dynamics, the roles of FGF21 and β-Klotho in muscle development, and the underlying mechanisms of muscle development need to be explored [132,138,139]. The reasons for this strange result may be numerous, but it also tells us something about the complexity and unpredictability of biological mechanisms. Moreover, this situation also provides excellent momentum for future research.
